# Deferred recovery of health expenditures for pediatric life-threatening emergencies in a resource-limited setting: Alternative before achieving universal health coverage in Cameroon in Central Africa

**DOI:** 10.1371/journal.pone.0322615

**Published:** 2025-06-05

**Authors:** Calixte Ida Penda, Charlotte Eposse Ekoube, Ritha Mbono Betoko, Cedric Nlend, Bertrand Eyoum Bilé, Francis Ateba Ndongo, Loic Boupda, Daniele Christiane Kedy Koum, Carole Eboumbou Moukoko, André Bita Fouda, Louis Richard Njock

**Affiliations:** 1 Faculty of Medicine and Pharmaceutical Sciences, University of Douala, Douala, Cameroon; 2 Laquintinie Hospital of Douala, Douala, Cameroon; 3 Douala General Hospital, Douala, Cameroon; 4 University of Garoua, Garoua, Cameroon; 5 Mother and Child Center, Chantal Biya Foundation, Yaounde, Cameroon; 6 Mokolo Regional Branch Hospital, Mokolo, Cameroon; 7 Deido District Hospital, Douala, Cameroon; 8 Pasteur Center of Cameroon, Yaoundé, Cameroon; 9 Ministry of Public Health, Yaounde, Cameroon; Federal Teaching Hospital, Ido-Ekiti, NIGERIA

## Abstract

The lack of health cover in low-income countries is a real barrier to emergency care. The objective of our study was to evaluate the immediate management of pediatric emergencies by deferred recovery of the costs of care at Douala Laquintinie Hospital. A prospective cross-sectional study was conducted from 1^st^ February to 30 June 2020 on patients admitted for life-threatening emergencies to the pediatric emergency department. Deferred recovery of healthcare costs was triggered by the issuance of a “green voucher, an internal reimbursement voucher issued by the doctor for expenses incurred upon patient admission in a life-threatening emergency and reimbursable within 72 hours after initial emergent management was received. Of the 786 patients admitted to the pediatric emergency department, 502 (63.8%) patients presented with a life-threatening emergency at a median age of 1 year [IQR: 0-5]. According to the indigence criteria, 40.4% of the patients were indigent and nearly 40% of the families’ patients declared having a monthly income < 50,000 franc of the French Colonies of Africa (FCFA) or 85 USD. The majority of patients with life-threatening 456 (90.8%) had benefited from the “green voucher” and 71.5% from care within 15 minutes of admission. The average household health expenditure during hospitalization was 143.9 ± 52.3 USD (53.5–393.9). A total of 76.1% of patients benefited from deferred care cost recovery, including 43.6% from moratorium payment facilities. The mortality rate was 9.8%. The deferred healthcare cost recovery system has proven effective in lowering avoidable child mortality in life-threatening emergencies, despite the heavy burden of healthcare costs for the underprivileged.

## Introduction

Universal health coverage (UHC) is defined as the possibility for all individuals to access to quality health care at an affordable cost [1]. Achieving UHC requires the development of efficient healthcare financing mechanisms. The main sources of funding are determined by three major groups: the state, health insurance (voluntary or compulsory) and direct payment for care by households. Direct healthcare cost recovery consists of payment of medical care fees and the purchase of essential drugs in hospital pharmacies [2]. Initially this health policy aimed to improve the care delivery and the allocation of resources, thus motivating the provision of services with a better quality/price ratio through community participation [3,4]. In Cameroon, the main method of payment for care is direct payment, which leads to fee-for-service payments for various services prior before to their execution [5]. In fact, one in thirteen Cameroonian children dies before the age of five (79 deaths per 1000 live births), a third of which during the first month of life (28 deaths per 1000 live births) [6]. These children’s deaths rate remain high compared to those occurring in rich countries where universal health care coverage or health insurance is widely used with better access and quality of care. Total health expenditure as a percentage of Gross Domestic Product (GDP) remained stable at around 4% from 2005 to 2017 in Cameroon, meaning an expenditure of 68 dollars per capita in 2017, significantly lower than the average in sub-Saharan Africa (86 dollars) [7]. In Cameroon the guaranteed minimum inter-professional wage was 36,270 FCFA (61.7 USD) in 2019, household funds constitute the main source of financing for the health sector, thus making emergency care inaccessible for the majority of the population [8]. Life-threatening emergencies (LTE) are characterized by the appearance of distress in one or more vital functions which can lead to death [9] and require rapid and adequate care without financial barriers. Previous studies carried out in the pediatric department at Douala Laquintinie Hospital (DLH) in 2013 and 2016 revealed a respective neonatal and infant mortality rate of 20.3% and 20.0% [10–12].

Furthermore, the epidemiological profile of LTE at DLH was that of a child under 5 years old, referred from a peripheral health establishment and presenting with a neurological emergency [12]. This high mortality rate gave rise to the implementation of a project to reduce neonatal and infant mortality in 2017 with the introduction of reforms in the operation of the service and the specific management of LTE. The aim of our work was to evaluate this immediate management of pediatric emergencies through the deferred recovery of care costs.

## Methods

### Study design and setting

A prospective cross-sectional study was carried out from 1^st^ February to 30^Th^ June 2020 in pediatric and newborn emergency care units of the DLH pediatric department. The DLH is a second category care and teaching hospital located in the city of Douala. The pediatric unit in DHL is managed by 5 pediatricians, 5 general practitioners and nurses.

#### Inclusion criteria.

All children and adolescents aged 0–19 years old admitted for life-threatening emergencies to the pediatric and neonatal emergencies services were included consecutively, voluntarily, anonymously and without remuneration after obtaining consent by the child’s parent or legal guardian.

#### Sample size calculation.

Assuming an estimated prevalence of children presenting with a pediatric emergency of 28.4% in Bamako, Mali in 2011 [13], the precision level ±5% and the confidence level 95%, the minimum sample size was 312 children using the Cochrane formula [14].


N = 1.96² x 0.284 x 0.716/0.05² = 312.4

### Study procedures

#### Management of the pediatric emergency service.

All patients were received in the emergency reception room where a rapid assessment of parameters and clinical condition were carried out for adequate triaging. Patient in a life-threatening emergency were admitted as soon as possible to the emergency room where conditioning and first aid were immediately performed. Ready-to-use emergency kits were available and comprised: blood transfusion kits, rehydration therapy, management of poisoning, lumbar puncture equipment, peripheral and central venous equipment, fever management and blood sampling kits for the rapid diagnostic test for malaria and HIV, blood sugar meters and urine dipstick. We transferred the patient to the appropriate unit as soon as he achieved clinical stability.

#### Technical procedures and conditions for issuing the internal care “green voucher”.

In the event of vital distress being diagnosed, a voucher for internal hospital care or “green voucher” was systematically issued by the doctor at reception. This voucher allowed immediate access without prior financial compensation to medications, blood products and urgent biological and radiological analyses, first aid was provided urgently after validation of the green voucher by the supervisor or on-call doctor of the various related services requested. The costs inherent in this voucher had to be reimbursed by the family within 48–72 hours. However, this period could be extended until discharge from the hospital in the event of proven indigence [15]. In the event of a request for a reduction of treatment costs due to the inability to pay the entire bill, payment facilities were granted by the hospital management after the establishment of a moratorium by the financial department.

An indigence score was produced for any children or adolescent admitted to the emergency room, based on criteria developed in the context of care of indigent HIV-infected children with the collaboration of the World Bank in 2006 and the Ministry of Social Affairs and adapted for the Department of Pediatrics in 2017 (Table 1). These criteria were based on the children’s rank among their siblings, the number of dependents, the number of children in school, the family situation, food difficulties, monthly income; the characteristics of the accommodation and its basic contents (home electricity, home drinking water, functional refrigerator, domestic gas, television).

**Table 1:** Identification of indigence [15].

Designation	Modalities
Number of children, n	1 to 2 = **1,** 3–5 = **2,** > 5 = **3**
Number of dependents, n	1 to 2 = **1,** 3–4 = **2,** > 4 = **3**
Family situation	NF = **1,** SPF/OF=**2,** OO/OM = **3**
Mode of management of the disease	Insurance/GV = **1** Species/ Family = **2**
Accommodation	Owner = **0,** Tenant in brick = **1,** Tenant in ground = **2** Plank house = **3,** living third party = **4**
Food difficulties	None = **0,** Monthly = **1,** Weekly = **2,** Daily = **3**
Monthly income in franc of the French Colonies of Africa (FCFA)	0 to 10,000 = **5,** 11,000–25,000 = **4** 26,000–50,000 = **3,** 51,000–75,000 = **2** 76 to 100,000 = **1,** > 100,000 = **0**
Home electricity	**0** = Yes, **1** = No
Home drinking water	**0** = Yes, **1** = No
Functional refrigerator	**0** = Yes, **1** = No
Gas cookers	**0** = Yes, **1** = No
TV	**0** = Yes, **1** = No
Computer	**0** = Yes, **1** = No
School children	**0** = Yes, **1** = No

**Legend.** NF: Normal family; SPF: Single parent family; OF: Fatherless; OO: Orphan of father and mother; OM: Motherless; GV: Green voucher; TV: television; 1franc of the French Colonies of Africa (FCFA): 0,0017USD

A form for determining the indigence score consisting of 14 items rated from 0 to 5 was systematically completed for each child and the sum of the points allowed us to classify each patient (Tables 1 and 2) [15].

**Table 2:** Scoring of the indigence score [15].

Designation	Indigence score	Degree of indigence	Family participation
	(n)	%	%
Notorious indigent	≥ 21	100	0
Permanent indigent	16–20	75	25
Medium needy	11–15	50	50
weak needy	6–10	25	75
None	≤ 5	0	100

• Was considered an indigent patient: any child with a score > 16.

• Was considered a needy patient: any child with a score > 6–15.

• Was considered a normal patient: any child with a score ≤5.

These criteria allowed us to determine the indigence score in the population studied according to Table 2.

### Data collection

Each patient admitted underwent a complete examination and an interrogation after the consent of the parent or legal guardian. Using a pre-established survey form, the data collected related to the following variables:

- The socio-demographic characteristics of the parents (marital status, level of education, profession) and of the patient (age, sex, origin);
- The classification of the indigence score;
- Clinical characteristics (medical and surgical history, previous treatment);
- Reason for consultation;
- Mode of entry (emergency, transfer from another hospital) and admission time;
- Vital parameters (temperature, heart rate, respiratory rate, oxygen saturation) and anthropometric parameters (weight, height, arm circumference);
- General signs (asthenia, anorexia, weight loss, mucocutaneous pallor, conscious cyanosis);
- The type of vital emergency (neurological; respiratory, cardio-circulatory);
- The time to therapeutic management for pediatric vital emergencies patients -
- The evolution of the patient (length of hospital stay; discharge against medical advice, healing, escapees, deceased).

### Statistical analysis

Sociodemographic, clinical, hospitalization course and financial data were collected using a pre-established questionnaire. Categorical variables were expressed as frequencies, while numeric variables were presented as means + /- standard deviation (SD). All statistical analyses were performed using Microsoft Excel 2016 and Statistical Package for Social Sciences (SPSS) version 20.0 software.

### Ethical consideration

The authorization of the Ethics Committee of the University of Douala n°2156/CEI-Udo/01/2020/T of January 21, 2020, was been obtained as well as the administrative authorization N° 00275/AR/MINSANTE/DHL/CM of January 09, 2020 of the DLH. Parents or legal representatives of participants were informed of the purpose and process of the survey (background, goals, objectives, methodology, data confidentiality, and rights to withdraw from the study without prejudice), and signed informed consent was obtained from parents/guardians of participants in accordance with the Declaration of Helsinki and verbal assent of all adolescents in accordance with national guidelines prior to study inclusion. The data was collected consecutively and anonymously with strict respect for human research.

## Results

### Hospital prevalence of pediatric life-threatening emergencies

**Fig 1:**
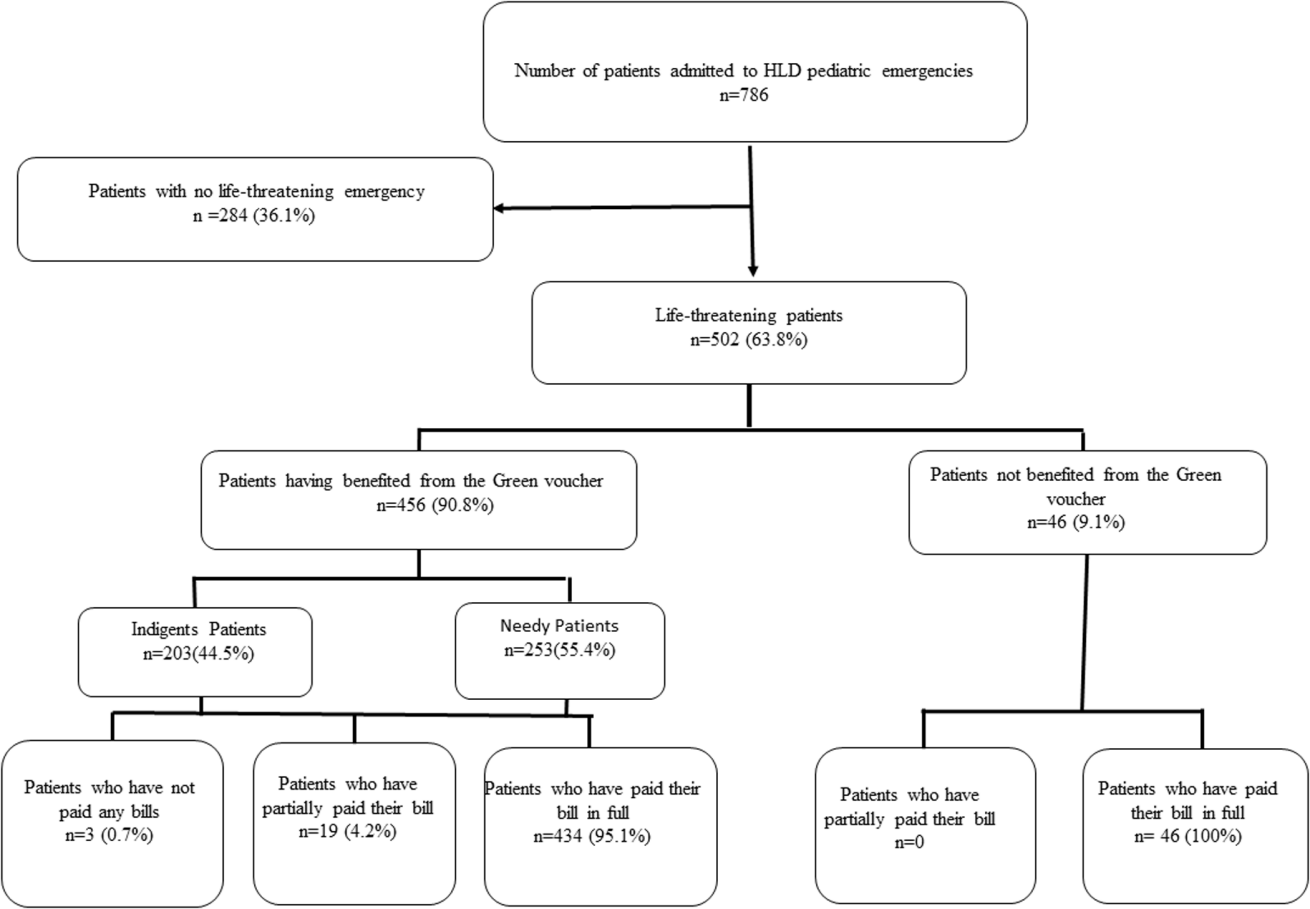
Flowchart of the study population in the pediatric emergency department of the Douala Laquintinie Hospital.

Of the 786 patients admitted to the pediatric emergency department of the DLH, 502 patients presented with a life-threatening emergency, i.e., a prevalence of 63.8% (Fig 1).

### Sociodemographic characteristics of the population studied

The male sex represented 54% and the sex ratio was 1.19. The median age was 1 [IQR: 0–5] years. Four out of five children came from a single-parent family. About half of the parents of the children had secondary education and 64.5% of the fathers of the patients worked in the informal sector (Table 3).

**Table 3:** Sociodemographic characteristics of the study population.

Variables	Number (n)	Percentage (%)
**Sex. n = 502**		
Male	274	54.7
Female	228	55.4
**Age (years)**		
0–5	382	76.1
6–9	43	8.5
10–15	54	10.8
16–19	23	4.6
**Marital status of parents**		
Married	101	20.1
Single	401	79.9
**Father’s level education**		
Primary	127	25.3
High school	247	49.2
University	101	20.1
Out of school	27	5.4
**Mother’s level education**		
Primary	137	27.0
High school	274	55.0
University	66	13.0
Out of school	25	5.0
**Father’s sector of activity**		
Formal	138	27.5
Unformal	324	64.5
Unemployed	40	8.0
**Mother’s sector of activity**		
Formal	59	11.8
Unformal	236	47.0
Unemployed	207	41.2

### Origin of patients in life-threatening pediatric emergencies according to the health district of residence and referring hospital

**Fig 2:**
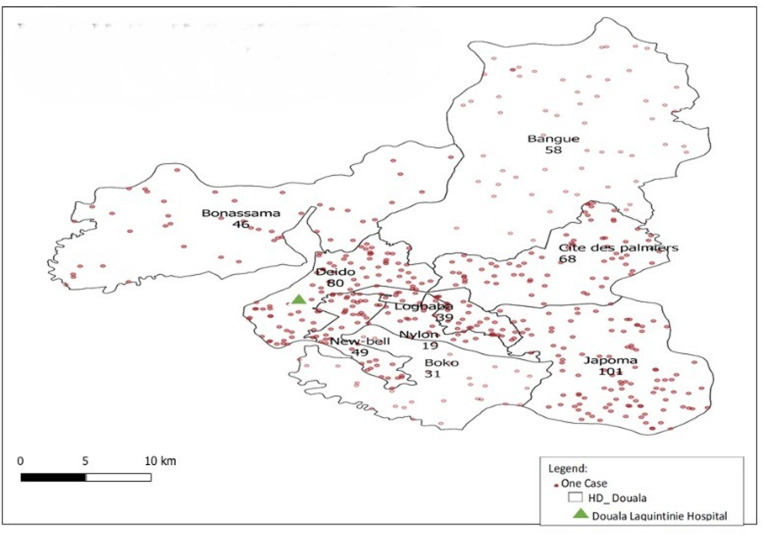
Origin of patients by health district of residence and hospital referral.

Only 80 (15.9%) of the patients resided in the DEIDO health district, to which the DLH belonged, while the rest came from other health districts, in particular that of JAPOMA (20.1%) and only 11 (2.2%) came from outside Douala (Fig 2).

Regarding the referring hospital, out of 502 patients with a life-threatening pediatric emergency (LTPE), more than a third 166 (33.1%) were referred from District medical centers/ Integrated health centers, 130 (25.9%) came directly from homes, 127 (25.3%) came from district hospitals and 79 (15.7%) from the Douala General Hospital and the DLH maternity service

### Determination of the indigence score

Large families with 3–5 children accounted for 48.6% of patients, 42.0% of families had 3–4 dependents and 32.5% were single parent families. Among the patients in life-threatening emergencies, 27.1% said they had difficulty getting the three daily meals during the month, 19.9% during the week and 12.7% on a daily basis. Nearly half of the families of the patients (45.2%) were tenants of houses made of permanent materials and 12.5% lived in houses made of temporary materials. All families had electricity, 48.8% had no running water supply and 48.2% lived without a functioning refrigerator. Nearly 40.0% of patients’ families declared having a monthly income of less than 85USD and nearly a third (31.1%) estimated their monthly income between 86.7 and 127.5USD (Table 4).

**Table 4:** Distribution of patients according to their family situation.

Variables	Number (n)	Percentage (%)
*Number of children*		
1–2	192	38.2
3–5	244	48.6
>5	66	13.1
*Number of dependents*		
1–2	152	30.3
3–4	211	42.0
>4	139	27.7
*Family situation*		
Couple	331	65.9
SPF/OF	163	32.5
OO/OM	8	1.6
*Accommodation*		
Tenant in permanent materials	227	45.2
Owner	138	27.5
Tenant in temporary materials	63	12.5
Living with third Parties	74	14.8
*Monthly income, USD*		
0–17	62	12.4
18.7–42.5	80	15.9
44.2–85	58	11.6
86.7–127.5	156	31.1
129.2–170	75	14.9
>170	71	14.1

**Legend.** SPF = Single parent family; OF = Fatherless; OM = Motherless; OO = Orphan of father and mother; 1Franc CFA (franc of the French Colonies of Africa) = 0.0017 USD (United State Dollars)

The distribution of patients according to the indigence score classified 203 (40.4%) patients as notorious indigent and 299 (59.4%) as needy indigent. The “green voucher”, internal care voucher was issued to 90.8% of patients in life-threatening emergencies (Fig 1).

### Patient care and evolution

The mean time to therapeutic management for pediatric vital emergencies (PVE) patients was 14 min.2sec ± 2 min.1sec (10–20 min) for 359 (71.6%). The average length of stay in pediatric emergencies was 6.06 ± 4.09 days (1–32). Among the patients admitted in a life-threatening emergency, 414 (82.5%) were declared cured and 49 (9.8%) had died.

Indigent patients belonging to families with 3–5 children accounted for 110 (54.2%) cases while families with (1–2) dependents were indigent in 26.1% of cases.

### Recovery of healthcare costs

The average health expenditure for children admitted to LTE was 143.9 ± 52.3 USD, of which for 329 (65.5%) patients, the amount varied between 86.7 and 170 USD and 1.2% of families had spent more than 340 USD. Out of 502 life-threatening patients, 490 (97.6%) of patients’ families had paid for their health care in cash. Nearly 40.0% of patients’ families declared having a monthly income < 85 USD and nearly a third (31.1%) estimated their income between 86.7 and 127.5USD.

The total cost of care to be recovered from PVE for the health facility represented 26,188.84USD during the study period, i.e., an average of 57.4 ± 21.31 USD per patient. The deferred cost recovery by the internal care voucher for 382 (76.1%) of the patients varied between 86.7–170 USD.

Half of the children admitted for vital emergencies 252 (50.2%) had benefited from payment facilities, including 219 (43.6%) from a moratorium and 33 (6.6%) from a reduction in costs to settle their bill.

Almost all of the needy 284 (97.6%) and 196 (92.9%) indigent patients had fully paid their bill on discharge (Table 5).

**Table 5:** Distribution of patients according to expenses.

Variables	Number (n)	Percentage (%)
***Health expenditure (USD)***		
≤ 85	22	4.4
86.7–170	329	65.5
171.7–255	137	27.3
256.7–340	8	1.6
≥ 340	6	1.2
***Method of payment***		
Insurance/Care voucher	12	2.4
Species/Family	490	**97.6**
***Amount of healthcare expenditure covered by Green voucher (USD)***		
≤ 85	46	9.2
86.7 - 170	**382**	**76.1**
171.7 - 255	74	14.7
***Payment facilities***		
***Moratorium (n = 502)***		
Yes	219	43.6
No	283	56.7
***Reduction in care costs (n = 43)***		
Reduction of 42.5USD	9	1.8
Reduction of 85USD	23	4.5
Reduction of 170USD	1	0.2

**Legend.** All values are in US Dollars; 1franc of the French Colonies of Africa (FCFA): 0.0017 USD

## Discussion

This study focused on the evaluation of the immediate management of pediatric emergencies by the deferred recovery of the costs of care at the DHL in Cameroon. At the end of this study, the hospital prevalence of vital pediatric emergencies was 63.8%. Nearly 3/5th of vital pediatric emergencies were of the neurological type and 90.8% of patients had benefited from the green voucher.

The median age was 12 months and the age group of children aged 0–5 years represented 76.1% of our patients. This result was close to that reported by several authors where the proportion of patients aged less than 5 years varied between 70.0% and 80.7% [12,16–18]. The great vulnerability due to immunological and functional immaturity could explain the high frequency of occurrence of LTE at this age. Boys were more affected, which was consistent with several previous studies conducted worldwide on pediatric emergencies where male gender were more susceptible to morbid phenomena in childhood [19,20].

In our study, 40.4% of the patients were notorious indigent on the basis of their social, family and financial conditions and 39.9% declared having a monthly income < 85 USD with a large family of 3–5 children (48.6%), 3–4 dependents (42.0%) and those with monthly food difficulties (27.1%) including 12.7% on a daily basis. In addition, 64.5% of fathers reported working in the informal sector, most often characterized by precarious activities generating low income. This precariousness has increased due to the health, socio-political and economic crises which could explain the difficulties of daily survival, additionally when a child is hospitalized in a context of vital distress. Indeed, Cameroon’s poverty rate remains high, stagnating around 40% since 2001, with an unemployment rate of 15.5% in 2014, then 6.1% in 2021, and the underemployment rate was estimated at 65.0% in 2021 [21,22]. In addition, the guaranteed minimum inter-professional wage (GMIPW) was 36,270 FCFA (60.4 USD) in 2014, and despite its revaluation in 2023–41,875 FCFA (69.7 USD) [8,23], it remained insufficient to cover health costs, including the average expenditure for households of children admitted for LTE were 84,647.8 FCFA (141USD).

The rate of issuance of the “green voucher” was 90.8% for patients admitted to LTPE and the mortality rate was 9.8%. We observed a 50% decrease in mortality compared to the results reported in 2021 in the same department where only 52.6% of PVE patients had received the green voucher with a LTPE mortality rate of 17.8% probably due to a better understanding of the community and hospital stakeholders [12]. Increasing the use of this device not only improves the prognosis of children with life-threatening emergencies by increasing the speed of treatment, but also restores a certain equity in the delivery of care. This thus contributes to the reduction of infant mortality by ensuring a fundamental right of the child.

The average time taken to take care of patients in life-threatening emergencies was 14 minutes, 2sec, of which 71.6% of patients were taken care of within 10–20 minutes. This early management of LTPE was linked to the delivery of the “green voucher” and the use of emergency kits which facilitated accessibility and improved the quality of care, unlike previous studies in a similar context where the minimum delay was 30 minutes in 4/6 children in Gabon and Nigeria [24,25]. Furthermore, the training of health personnel in the early identification of LTE is another important step in improving care, as shown by several studies [26–28].

The average household health expenditure during hospitalization was 141USD (52.4–385.9) and ranged from 84.9 to 166.5 USD for 65.5% of patients. A total of 76.1% patients benefited from deferred care cost recovery, including 43.6% from payment facilities in the form of a moratorium. In our context, cost recovery in public health facilities remains a significant concern due to numerous cases of insolvency [29], in addition very few Cameroonians are covered by a social health protection mechanism. The level of household health expenditure (68%) remains high and well above the WHO standard (15–20%), the average for countries in sub-Saharan Africa (33%) and countries with similar incomes such as than Kenya (24%) and Ghana (40%) [1,7].

The payment facilities granted to patients thus enabled low-income households to pay their debt while continuing to meet the daily needs of their families and the health unit to recover almost all the costs by surrounding itself with a minimum guarantees concluded between the two parties.

The total cost of care to be recovered from LTPE for the health facility was 26,188.8USD during the study period, meaning an average of 57.4 + 21.31USD per patient. The non-payment of the costs of care could have a negative impact on the functioning of the service, patients’ prognosis and the hospital management. This can lead to a disruption in the payment of debts owed to suppliers of inputs, consumables and essential drugs, as well as a reduction in staff motivation bonuses which contribute to improving the quality of care. UHC could therefore constitute a support strategy for the most vulnerable groups who generally experience difficulties in paying for healthcare.

## Conclusion

The hospital prevalence of LTPE was high and indigent patients represented almost half of the study population. The average health expenditure on first aid for LTPE was higher than the GMPIW in Cameroon. The use of the internal care “green voucher” made it possible to accelerate patient care and to have a favorable prognosis for the majority of patients. The deferred care cost recovery system has proven effective in reducing infant mortality in life-threatening emergencies despite the costs of health care borne by families, particularly those who were disadvantaged.

### Limit of the study

This study only considers the direct costs of care, but hospitalization also generates other indirect costs. Transportation, meals, loss of income due to suspension of activities and other non-exhaustive indirect costs associated with hospitalization.

The study was carried out in a single center.

### What this study brings

- The evaluation of an innovative health financing strategy in emergency situations, adapted to the context of limited resources and which can serve as an alternative to the direct payment required by the act.
- The average cost of health expenditures in pediatric emergencies
- This strategy can be transitional and support the process of progressive implementation of full universal health coverage.

## Supporting information

S1 File Codified database. (XLSX)

S2 File Key of interpretation. (CSV)

S3 File Base_new. (CSV)

## Abbreviations

UHC: universal health coverage
LTE: life-threatening emergencies
GDP: gross domestic product
DLH: Douala Laquintinie Hospital
LTPE: life-threatening pediatrics emergencies
NF: normal family
SPF: single parent family
OF: fatherless
OO: orphan of father and mother
OM: motherless
GV: green voucher
TV: television
SPF: single parent family
WHO: World Health Organization
USD: US Dollar
CFA franc: franc of the French Colonies of Africa.
